# Supplementary material for: Integrating mindfulness and physical activity: a meta-analysis of mindful movement interventions for symptoms of anxiety and depression among university students
Source: PeerJ. 2025 Jul 1;13:e19640. doi: 10.7717/peerj.19640 (PMC12226987; doi:10.7717/peerj.19640)
Supplement: Supplemental Information 1 [file peerj-13-19640-s001.docx]

Table S1 The search strategy of literature

| Library | search strategy | Number |
| --- | --- | --- |
| PubMed | ((((("Students"[Mesh]) OR ("Young Adult"[Mesh])) OR ((((university student[Title/Abstract]) OR (college student[Title/Abstract])) OR (undergraduate student[Title/Abstract])) OR (freshman[Title/Abstract]))) AND (((("Mindfulness"[Mesh]) OR ("Exercise"[Mesh])) OR ("Movement"[Mesh])) OR (((((((((Mindful Movement[Title/Abstract]) OR (Yoga[Title/Abstract])) OR (Tai Chi[Title/Abstract])) OR (Qigong[Title/Abstract])) OR (Mindful Walking[Title/Abstract])) OR (Physical Activity[Title/Abstract])) OR (Aerobic Exercise[Title/Abstract])) OR (Exercise Trainings[Title/Abstract])) OR (Resistance Training[Title/Abstract])))) AND ((((((("Mental Health"[Mesh])) OR (((Health, Mental[Title/Abstract]) OR (Mental Hygiene[Title/Abstract])) OR (Hygiene, Mental[Title/Abstract]))) OR ("Anxiety"[Mesh])) OR ((((Angst[Title/ Abstract]) OR (Nervousness[Title/Abstract])) OR (Hypervigilance[Title/Abstract])) OR (Anxiousness[Title/Abstract]))) OR ("Depression"[Mesh])) OR (((Depressive Symptoms[Title/Abstract]) OR (Depressive Symptom[Title/Abstract])) OR (Emotional Depression[Title/Abstract])))) AND (((Randomized controlled trial[Title/ Abstract]) OR (randomized[Title/Abstract])) OR (placebo[Title/Abstract])). | 383 |
| Cochrane | #1 MeSH descriptor: [Students] explode all trees #2 (Unversity student):ti,ab,kw or (College student):ti,ab,kw or (undergraduate student):ti,ab,kw or (freshman):ti,ab,kw #3 MeSH descriptor: [Young Adult] explode all trees #4 #1 or #2 or #3  #5 MeSH descriptor: [Mindfulness] explode all trees #6 MeSH descriptor: [Exercise] explode all trees  #7 MeSH descriptor: [Movement] explode all trees #8 (Mindful Movement):ti,ab,kw or (Yoga):ti,ab,kw or (Tai Chi):ti,ab,kw or (Qigong):ti,ab,kw or (Mindful Walking):ti,ab,kw or (Physical Activity):ti,ab,kw or (Aerobic Exercise):ti,ab,kw or (Exercise Trainings):ti,ab,kw or (Resistance Training):ti,ab,kw #9 #5 or #6 or #7 or #8 #10 MeSH descriptor: [Mental Health] explode all trees #11 MeSH descriptor: [Anxiety] explode all trees #12 MeSH descriptor: [Depression] explode all trees #13 (Health, Mental):ti,ab,kw or (Mental Hygiene):ti,ab,kw or (Hygiene, Mental):ti,ab,kw or (Angst):ti,ab,kw or (Nervousness):ti,ab,kw or (Hypervigilance):ti,ab,kw or (Anxiousness):ti,ab,kw or (Depressive Symptoms):ti,ab,kw or (Depressive Symptom):ti,ab,kw or (Emotional Depression):ti,ab,kw #14 #10 or #11 or #12 or #13 #15 #4 and #9 and #14 | 1067 |
| Web-Sci | 1 ((((((TI=(students)) OR TI=(university student)) OR TI=(College student)) OR TI=(undergraduate student)) OR TI=(freshman))  OR TI=(young adult)) NOT (SILOID==("PPRN")) 2 (((((((((TS=(Mindfulness)) OR TS=(Mindful Movement)) OR TS=(Yoga)) OR TS=(Tai Chi)) OR TS=(Qigong)) OR TS=(Mindful Walking)) OR TS=(Exercise)) OR TS=(Aerobic Exercise)) OR TS=(Exercise Trainings)) OR TS=(Resistance Training) and Preprint Citation Index (Exclude – Database) 3 (((((TS=(Mental Health)) OR TS=(Health, Mental)) OR TS=(Mental Hygiene)) OR TS=(Hygiene, Mental)) OR TS=(anxiety)) OR TS=(Depression) and Preprint Citation Index (Exclude – Database) 4 ((TS=(Randomized controlled trial)) OR TS=(randomized)) OR TS=(placebo) and Preprint Citation Index (Exclude – Database) #1 AND #2 AND #3 AND #4 and Preprint Citation Index (Exclude – Database) | 475 |
| Embase | #3 AND #7 AND #12 AND #13 'randomized controlled trial':ti,ab OR 'randomized':ti,ab OR 'placebo':ti,ab  #8 OR #9 OR #10 OR #11 'health, mental':ti,ab OR 'mental hygiene':ti,ab OR 'hygiene, mental':ti,ab OR 'angst':ti,ab OR 'nervousness':ti,ab OR 'hypervigilance':ti,ab OR 'anxiousness':ti,ab OR 'depressive symptoms':ti,ab OR 'depressive symptom':ti,ab OR 'emotional depression':ti,ab OR'depression'/exp OR 'anxiety'/exp OR'mental health'/exp  #4 OR #5 OR #6. 'mindful movement':ti,ab OR 'yoga':ti,ab OR 'tai chi':ti,ab OR 'qigong':ti,ab OR 'mindful walking':ti,ab OR 'physical activity':ti,ab OR 'aerobic exercise':ti,ab OR 'exercise trainings':ti,ab OR 'resistance training':ti,ab OR 'exercise'/exp OR'mindfulness'/exp  #1 OR #2. 'university student':ti,ab OR 'college student':ti,ab OR 'undergraduate student':ti,ab OR 'freshman':ti,ab OR 'young adult':ti,ab 'student'/exp | 261 |
