# Supplementary figures and images for: Integrating mindfulness and physical activity: a meta-analysis of mindful movement interventions for symptoms of anxiety and depression among university students
Source: PeerJ. 2025 Jul 1;13:e19640. doi: 10.7717/peerj.19640 (PMC12226987; doi:10.7717/peerj.19640)

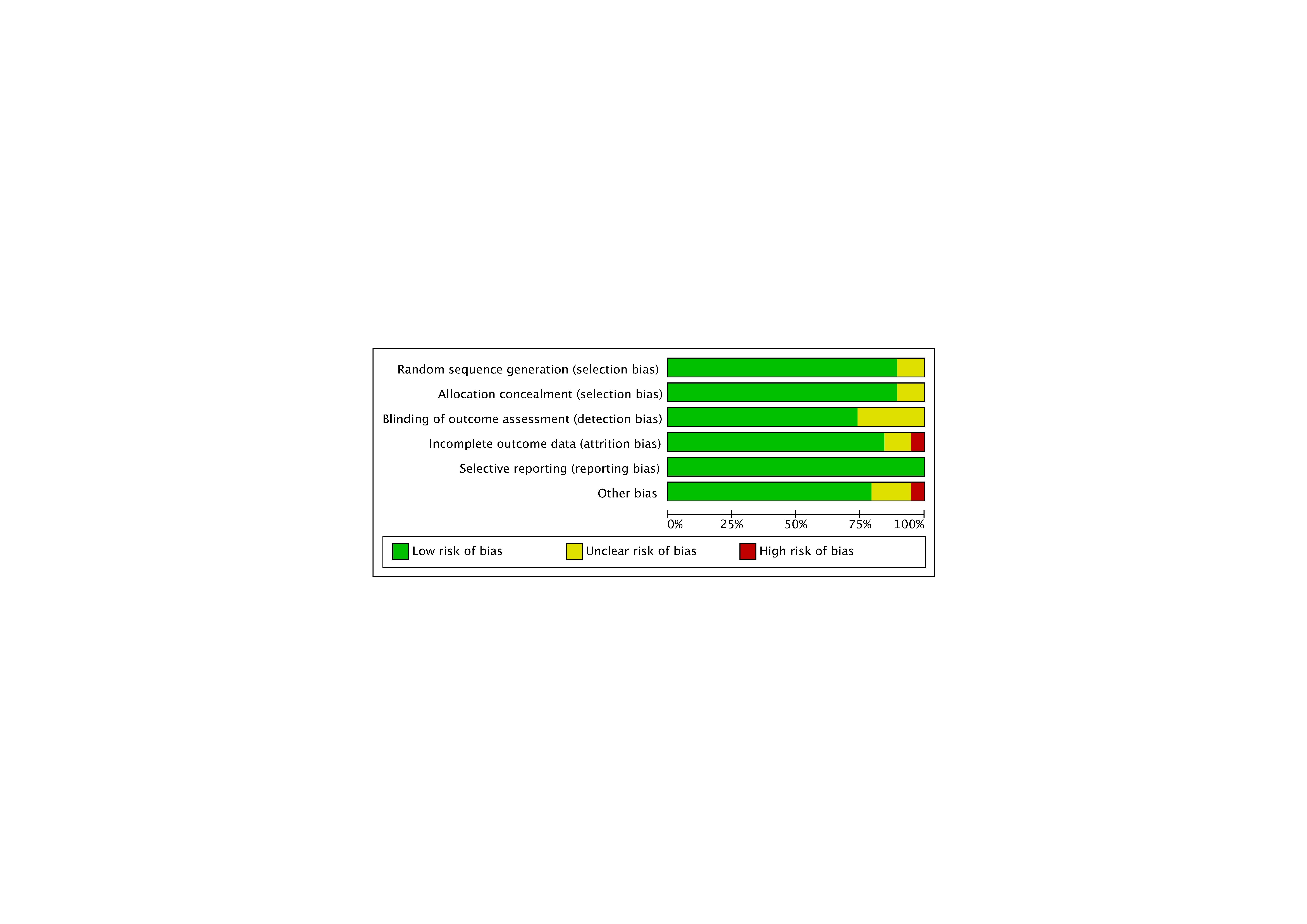

Supplement: Supplemental Information 2 [file peerj-13-19640-s002.png]

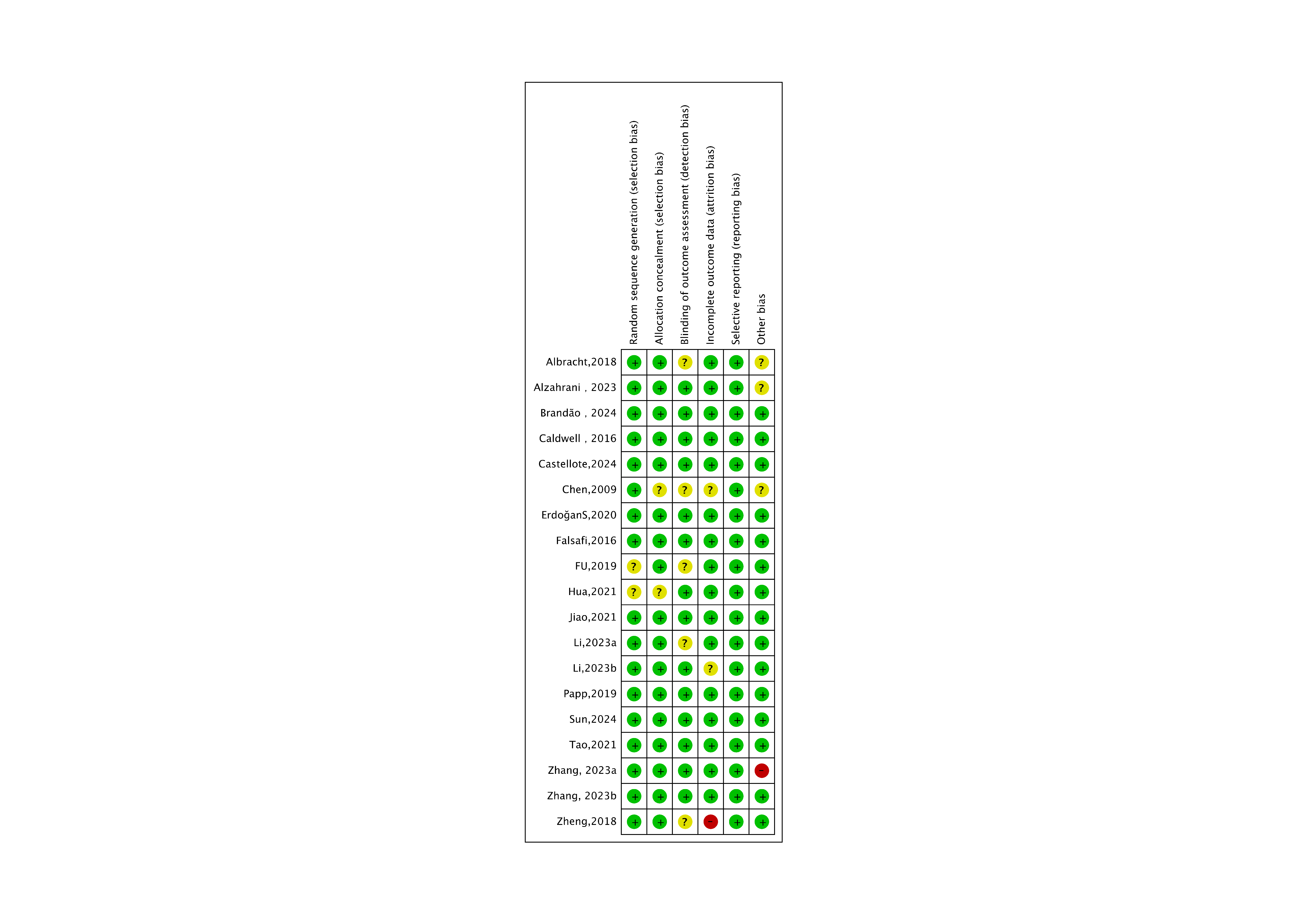

Supplement: Supplemental Information 3 [file peerj-13-19640-s003.png]

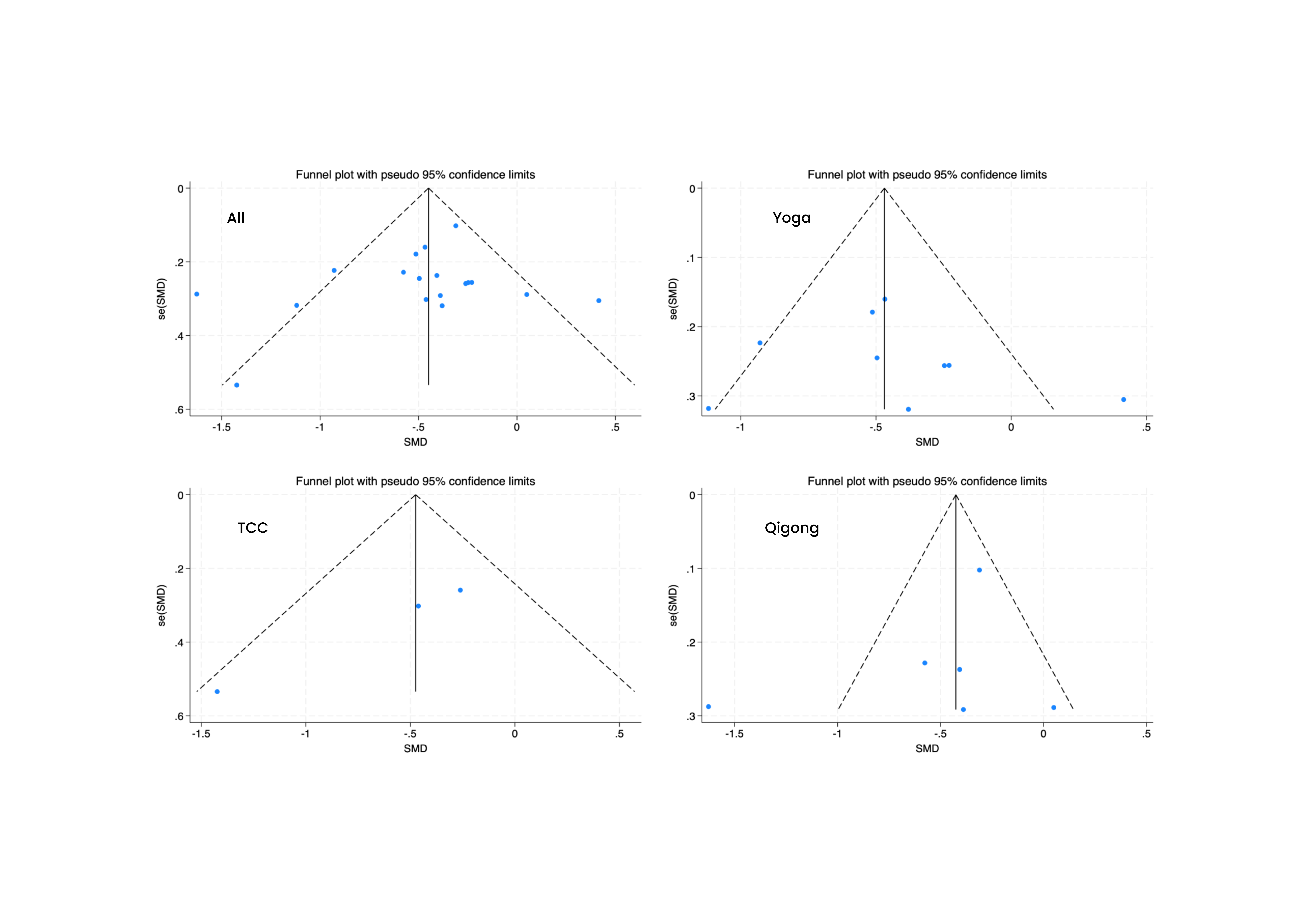

Supplement: Supplemental Information 4 [file peerj-13-19640-s004.png]

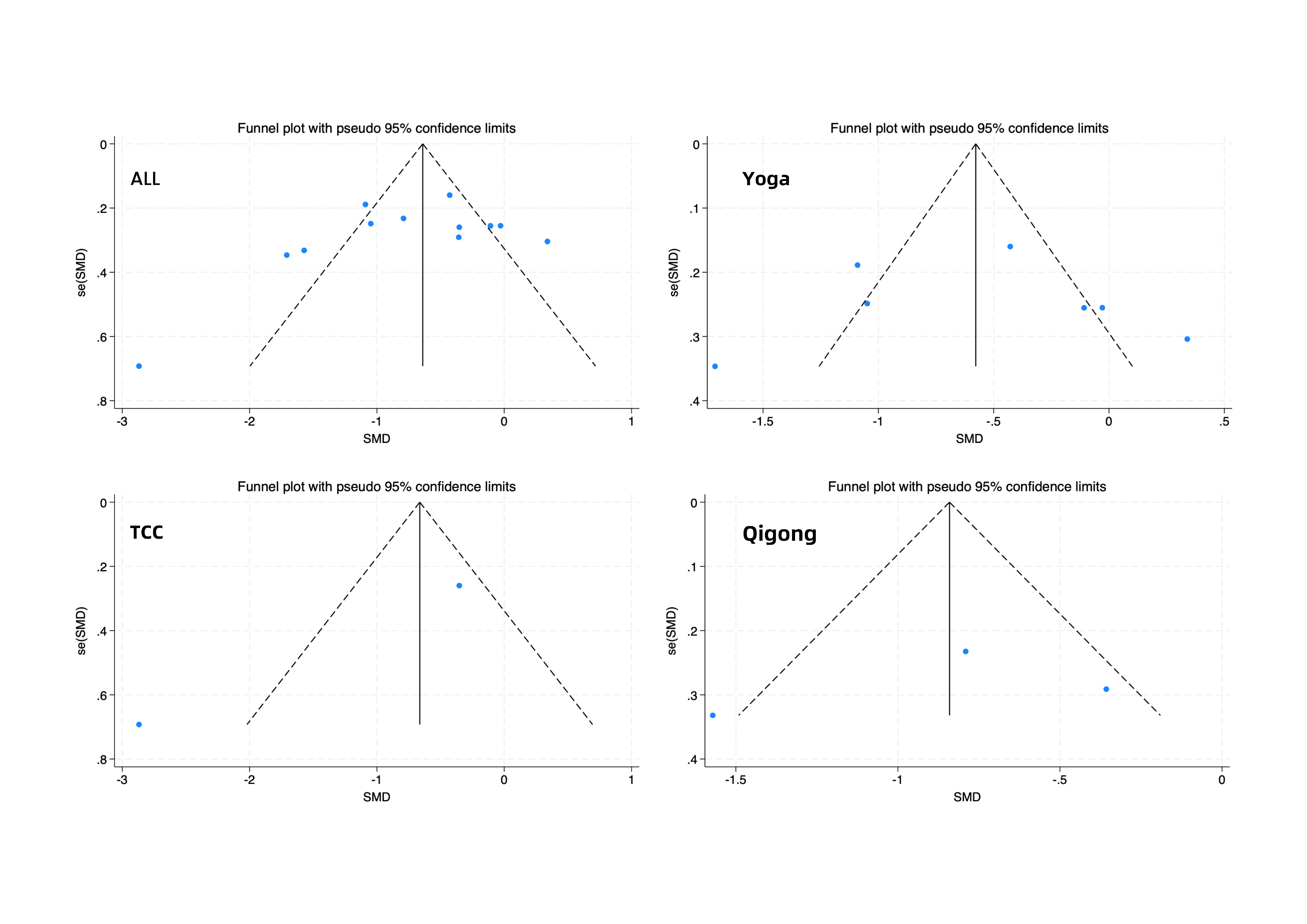

Supplement: Supplemental Information 5 [file peerj-13-19640-s005.png]
